# Culture and the assumptions about appearance and reality: a scientometric look at a century of research

**DOI:** 10.3389/fpsyg.2023.1140298

**Published:** 2023-09-15

**Authors:** Alessandro Carollo, Alfonso Maria Stanzione, Seraphina Fong, Giulio Gabrieli, Albert Lee, Gianluca Esposito

**Affiliations:** ^1^Department of Psychology and Cognitive Science, University of Trento, Rovereto, Italy; ^2^School of Social Sciences, Nanyang Technological University, Singapore, Singapore

**Keywords:** scientometric, systematic review, appearance, reality, appearance vs. reality, document co-citation, CiteSpace

## Abstract

**Introduction:**

People represent the world in terms of two constructs: how something appears on the surface (appearance) and what it is underneath that surface (reality). Both constructs are central to various bodies of literature. What has not been done, however, is a systematic look at this collection of literature for overarching themes. Motivated by this research gap, the present scientometric review aimed to identify the common themes that penetrate through a century of scholarly work on appearance and reality. In doing so, this review also sketched a scientometric outline of the international network, pinpointing where the work was carried out.

**Methods:**

With CiteSpace software, we computed an optimized document co-citation analysis with a sample of 4,771 documents (1929–2022), resulting in a network of 1,785 nodes.

**Results and discussion:**

We identified impactful publications, summarized major intellectual movements, and identified five thematic clusters (“Perception of Counseling Services”, “Appearance and Reality in Sociocultural Evolution,” “Cultural Heritage and Identity,” “Media and Culture,” and “Cultural Identity”), all with theoretical and pragmatic implications which we discuss. A deeper look at these clusters reveals new empirical questions and promising directions for future research.

## Culture and the assumptions about appearance and reality: a scientometric approach

People represent the world in terms of two constructs: how something appears on the surface (appearance) and what it is underneath that surface (reality). The relationship between appearance and reality has been the object of philosophical debate since Greek philosophy (Moravcsik, 1991). A crucial point in such philosophical debate occurred in the nineteenth century when Schopenhauer discussed the separation of the world as it appears (appearance) from the world as it is (reality) through the concept of the veil of Maya (Russell, 1912; Gardner, 2015; Schopenhauer, 2020). In his seminal work *Problems of Philosophy*, Russell (1912) illustrated the distinction between appearance and reality with an example: The table which can be seen and touched (appearance) is not the same as the underlying nature of the table (reality), which may consist of atoms, particles, and other elements that are not directly perceptible through vision or the sense of touch.

Similar discussions have flourished on the other side of the world. For instance, the concept “appearance” is known as *xiang* (象) in classical Chinese, whereas the concept “reality” is *benxiang* (本相), literally translated as “the original states of things.” In Japan, the line that separates appearance from reality is manifested in the distinction between tatemae (建前), which literally means “facade” or “front,” and honne (本音), the “true sound” or “the inner reality.” Appearance, xiang, honne, all these terms fall under the same conceptual umbrella, denoting the visible manifestations of what is invisible (e.g., reality, benxiang, tatemae).

In some fields of psychology, the association between appearance and reality has been conceptualized as two lay theories: convergence and divergence theories of appearance (Lee et al., 2020). On the one hand, convergence theory, in its core form, assumes that appearance is an accurate reflection of reality. Inferences guided by convergence theory tend to follow the rule of “what you see is what you get,” be it an inference about people, objects, or events. For instance, with convergence theory, people may be inclined to infer that someone who looks intelligent or happy on the outside is indeed intelligent or happy on the inside. Inferences suggesting the use of convergence theory are well-documented in the psychology literature (e.g., Traub, 1983; Paulhus and Morgan, 1997; Zebrowitz et al., 2002; Cogsdill et al., 2014; Charlesworth et al., 2019), some of which are traceable to the what-is-beautiful-is-good stereotype (Dion et al., 1972). While convergence theory can be observed around the world, it has been historically prevalent in the West as a basic assumption that people use for a variety of social judgments, such as opponent evaluations in a competition (Lee et al., 2020). On the other hand, divergence theory, in its core form, assumes that appearance is a misleading reflection of reality. Inferences guided by divergence theory tend to follow the rule of “things are not how they seem,” with implications for a host of social judgments. While divergence theory is not exclusive to any culture, it has been historically embraced by the East as an underlying assumption about the world (Lee et al., 2020; Ji et al., 2023). For instance, research suggests that East Asians are more prone to infer that the inner state of things deviates from their outward appearance when making inferences about people or events (e.g., Choi and Nisbett, 2000; Maddux and Yuki, 2006; Masuda et al., 2008; Lee et al., 2020; Ji et al., 2023). In fact, the contrast of appearance and reality cuts so deep into Chinese culture to the point it has left a mark in the realm of philosophy (e.g., in Chinese culture, sophistication on the inside is often framed in the appearance of flaws and foolishness, or “大智若愚,” literally translated as “the highest wisdom looks like foolishness”).

In many ways, convergence and divergence theories of appearance form the foundation of numerous life domains. For example, appearance and reality are central to the field of epistemology, which has to do with the nature and limitation of human knowledge. Identifying the relationship between appearance (how things seem to be) and reality (what they truly are) helps to answer big questions such as the extent to which human perception is reliable or whether beliefs are valid (e.g., Russell, 1912). Another example can be drawn from perception and cognition. The way people interpret sensory information is influenced by whether they believe that sensory information is a straightforward reflection of reality or not (e.g., Paulhus and Morgan, 1997; Zebrowitz et al., 2002; Cogsdill et al., 2014; Charlesworth et al., 2019; Lee et al., 2020; Ji et al., 2023). Distinct theories about appearance also have implications for decision-making processes by shaping the way people make sense of situations, assess risks, and evaluate possible outcomes (e.g., Maddux and Yuki, 2006; Spina et al., 2010). Other examples emerge from domains of cognitive development (Taylor and Flavell, 1984), literature and art (Schreiner, 2003), politics (Connolly, 1981), and more (e.g., Messaris, 1997; Hellman, 2001; Simon-Kerr, 2020). This body of literature points to distinct views on appearance and reality, which vary across people and cultures. Complementing this work, the current work aims to systematically examine the patterns of international collaborations and the trends in research in the whole literature of the field. Particularly, a scientometric approach was adopted to investigate the literature on the assumptions about appearance and reality. Scientometrics is an approach that merges scientific mapping (i.e., visualization of the evolution of a domain of research over time) and bibliometric analysis (i.e., the use of quantitative techniques on bibliometric data) (Nakagawa et al., 2019; Donthu et al., 2021; Sabe et al., 2022). Scientometric reviews are showing promising results in several fields across psychology, neuroscience, and engineering (Carollo et al., 2021a; Sood et al., 2022, 2023a, 2023b; Kumar et al., 2023). Specifically, in the current work, we aimed to: (i) identify the most active countries in the research field and the pattern of international collaboration (Belli and Baltà, 2019); (ii) identify the impactful documents; and (iii) identify the major thematic developments. The scientometric approach chosen in the present review allows for a data-driven systematic organization of the literature of interest. Our goal is to shed light on the common themes that revolve around the assumptions about appearance and reality and, in the process, identify promising directions for future research. In this way, the current study aims to provide insight on to the role of culture in shaping people’s perception about reality in a systematic and data-driven fashion.

## Methods

### Materials

The current work adopted the scientometric methodology of previously published reviews in the field of neurobiology, social neuroscience, and clinical psychology (Carollo et al., 2021a,b,c). In line with previous publications on scientometric frameworks (Jayantha and Oladinrin, 2019), all data for the current review were downloaded from the Scopus platform. Scopus was chosen over other platforms (e.g., Web of Science) for its wider coverage of indexed journals and availability of documents (Cataldo et al., 2022; Lim et al., 2023a). In order to include the largest number of articles, the following series of topic specific keywords for the bibliographic research was used: “TITLE-ABS-KEY (cultur* OR ‘cultur* think*’ AND (*appear* OR *real*) AND assumpt*) AND (LIMIT-TO (LANGUAGE, ‘English’)).” These key terms were optimized in order to retrieve the literature of interest. This data search led to a sample of 4,771 documents, covering a period of time from 01 January 1929 to 31 December 2022. All documents were collected from Scopus on 30 January 2023. The sample of data included 2,908 journal articles, 889 book chapters, 345 reviews, 292 books, 261 conference papers, 41 editorials, 18 notes, 5 short surveys, 4 letters, and 2 conference reviews. No restriction on document types and document content was applied when retrieving data in order to maximize the sample of data for the data-driven analysis, following the approach of previously published scientometric reviews (e.g., Bonacina et al., 2023; Lim et al., 2023a). To investigate the collaborations across countries, the downloaded sample of documents was initially analyzed with *bibliometrix* package for R (Aria and Cuccurullo, 2017; Lim et al., 2023a).

Data were subsequently imported into CiteSpace (version 6.1.R6), which is the software chosen to conduct the scientometric analysis (Chen, 2006; Ruan and Yan, 2022). CiteSpace was chosen because it allows identifying and visualizing the main thematic domains of research based on the patterns of co-citations among documents. When importing the data, the software identified 282,591 references cited in the papers retrieved from Scopus. Among the total number of references, 263,346 were considered valid (93.19%). When importing data into CiteSpace, small amounts of invalid references depend on data irregularities in the downloaded data pool (Chen, 2016; Carollo et al., 2021a). Thus, the data loss of the current study (*n* = 19,245; 6.81% of the total number of identified references) can be considered negligible (Gaggero et al., 2020).

### Document co-citation analysis

As the interest of the current scientometric review was to examine impactful publications and research domains in the study of culture and the assumptions about appearance and reality, a document co-citation analysis (DCA) was computed (Hou et al., 2018; Trujillo and Long, 2018; Neoh et al., 2023a). With the DCA, a network of relevant documents emerges from the analysis of the co-citation patterns between documents, i.e., the frequency in which multiple documents are cited together by subsequent documents (Small, 1980; Carollo et al., 2021a). Thus, the resulting network depends on both the citing papers (the ones directly downloaded from Scopus) and the documents cited by them (identified when importing data into CiteSpace; Chen, 2014; Aryadoust et al., 2021). This type of analysis is grounded on the assumption that clusters of papers frequently cited together represent thematic trends of research in the specific scientific domain of interest (Carollo et al., 2021a,b; Lim and Aryadoust, 2021; Lim et al., 2023b). In CiteSpace, DCA is created by modeling documents as single nodes and their co-citation as links. Furthermore, the frequencies of co-citation, which are measured in terms of cosine coefficients, are modeled as link weights. Based on the links weights, the CiteSpace’s “Find cluster” function identifies thematic trends of research. To do so, CiteSpace uses a hard clustering approach to divide the network into non-overlapping clusters (Chen et al., 2010). When identified, clusters are automatically labeled by CiteSpace using one of the labeling methods available. For the current work, all labels were initially generated using the log-likelihood ratio (LLR) approach. The LLR approach extracts noun phrases from the titles and abstracts of the citing documents and use them to reflect a unique aspect of a cluster. LLR was chosen because it provides the most accurate labels as compared to other methods for automatic cluster labeling. However, clusters’ labels were manually fixed when the LLR labels lacked accuracy based on the qualitative inspection of the citing documents included in the clusters (as in Carollo et al., 2023).

A DCA’s network is generated by choosing the node selection criterion of interest and setting its scale factor. The node selection criterion determines the strategy that is used by the software to determine whether a document should be included in the final network or not. The scale factor, on the other hand, is a numeric value that sets the threshold for the adopted node selection criterion. To adopt the optimal node selection criterion with the optimal scale factor, we compared the results obtained by computing several DCAs.

**FIGURE 1:**
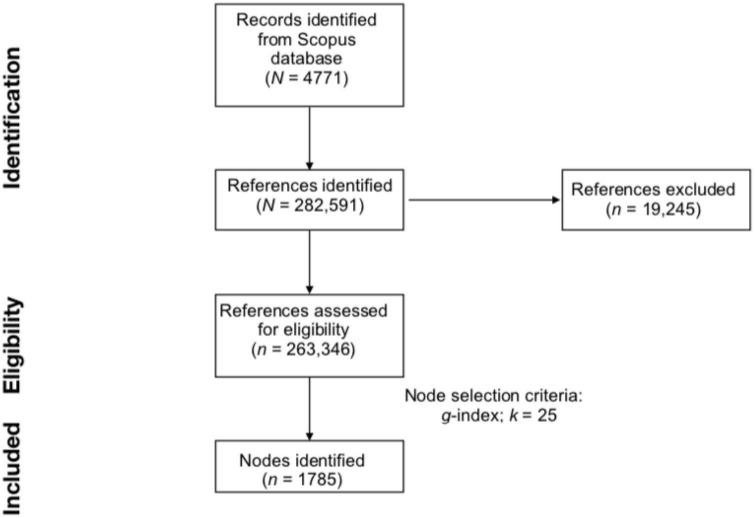
Study flow diagram. The sample of publications were collected from Scopus using the keywords “TITLE-ABS-KEY (cultur* OR ‘cultur* think*’ AND (*appear* OR *real*) AND assumpt* AND (LIMIT-TO (LANGUAGE, ‘English’)).” Data were collected on 30 January 2023.

In particular, three selection criteria were adopted: g-index, Top N, and Top N%. G-index is an improvement of the h-index and represents the “largest number that equals the average number of citations of the most highly cited g publications” (Egghe, 2006; Alonso et al., 2009; Chen et al., 2010). When setting the scaling factor *k* to 1, the standard g-index is used. Conversely, higher values of *k* correspond to a higher number of included documents (Neoh et al., 2023b). TOP N includes all the N most cited documents within a time slice into the network. Similarly, TOP N% includes all the N% most cited documented within a time slice into the network. For instance, when *N* is set to the value of 25, the TOP N criterion will include in the network the 25 most cited documents by time slice. Conversely, with the same thresholding value the TOP N% criterion will include the 25% most cited documents in each time slice. For the current study, the time slice was always maintained at a value of 1 year (Chen, 2014). DCAs generated by using g-index with k set at 25 and 50, TOP N with N set at 50, TOP N% with N set at 5 and 10 were tested and compared. All these thresholding values were tested after generating a network with the default scaling factors. After generating a DCA with the default values, subsequent scaling factor thresholds were selected in the attempt to optimize the metric of the network by being more liberal or stringent with the number of nodes included [as indicated in the CiteSpace manual by Chen (2014)]. In order to be as comprehensive as possible, the “Look back years” parameter was always set at −1. In this way, all the cited references within a citing document were analyzed to construct the network regardless of their temporal distance from the citing paper (Gaggero et al., 2020). The resulting DCAs were compared in terms of overall structural configuration (i.e., number of nodes, links and clusters, modularity Q, and silhouette score). The node selection criterion that optimized the structural features of the network was g-index with k set at 25. The procedure is summarized in Figure 1.

### Metrics

In agreement with standard scientometric works (e.g., Chen et al., 2010), the results from CiteSpace are described in terms of structural and temporal metrics.

Structural metrics include modularity Q, silhouette score, betweenness centrality. Modularity Q, with values ranging from 0 to 1, indicates the degree to which it is possible to divide the network into single groups of nodes, i.e., thematic clusters of research (Newman, 2006). High values of modularity Q are indexes of a well-structured network, which can be divided into thematic cluster of research (Chen et al., 2010). Conversely, silhouette score, with possible values ranging from −1 to 1, measures the inner consistency (i.e., cohesion and separation) of the identified clusters (Rousseeuw, 1987). Larger values are obtained by clusters that are strongly separated from others as well as the ones having a high inner consistency (Chen et al., 2010; Chen and Song, 2019). In the current work, higher values of silhouette scores will be obtained by thematic clusters of research that are highly consistent and separate from other clusters of research. Lastly, betweenness centrality defines the degree to which a single node can function as a bridge in connecting an arbitrary pair of nodes in the network (Freeman, 1977; Chen, 2014). Centrality values range from 0 to 1 and higher values are typically obtained by revolutionary scientific works that connect two otherwise unrelated clusters of research (Aryadoust et al., 2019).

The group of temporal metrics includes citation burstness and sigma. Citation burstness indicates an abrupt increase in the number of citations received by a document in a given time frame (Chen, 2017). Values of citation burstness are computed through Kleinberg’s algorithm (Kleinberg, 2003). In the current manuscript, citation burstness will be an index of the documents’ impact in the field of interest. Lastly, sigma scores reflect the novelty of a document and its impact on the overall network (Carollo et al., 2021a). Sigma values are computed by combining scores of centrality and burstness using the following equation: (centrality + 1)^burstness^ (Chen et al., 2009).

## Results

The DCA resulted in a network composed of 1,785 nodes and 4,708 links. Therefore, on average, each node (i.e., each reference) included in the network was connected to 2.63 other nodes. The generated network resulted to be well-structured and highly divisible into discrete clusters (modularity *Q* = 0.9008). On average, clusters in the network had high internal consistency and were well separated from other clusters (silhouette score = 0.9053).

In the network, 11 nodes had a citation burst in their history (see Table 1 for the complete list of references having a citation burst). Specifically, the document reporting the strongest citation burst was published by Bourdieu (1984) (strength of burstness = 9.27; duration of burstness = 9 years), followed by the works authored by Butler (1990) (strength of burstness = 8.15; duration of burstness = 6 years) and Beck et al. (1992) (strength of burstness = 7.54; duration of burstness = 6 years). Documents also varied in terms of duration of their citation burst. Specifically, two documents displayed the longest burst duration: Bourdieu (1977) (duration of burstness = 11 years; strength of burstness = 4.97) and Anderson (1983) (duration of burstness = 10 years; strength of burstness = 7.20).

**TABLE 1:** Identifying characteristics of the 11 references that had a citation burst within the computed DCA network.

References	Strength of burstness	Year	Beginning of burstness	End of burstness	Burst duration	Centrality	Sigma
Bourdieu, 1984	9.27	1984	2013	2022	9	0.01	1.09
Butler, 1990	8.15	1990	2010	2016	6	0.02	1.13
Beck et al., 1992	7.54	1992	2007	2013	6	0.02	1.18
Anderson, 1983	7.20	1983	2008	2018	10	0.03	1.25
Geertz, 1973	5.99	1973	2009	2013	4	0.03	1.16
Goffman, 1959	5.46	1959	2016	2019	3	0.01	1.03
Appadurai, 1996	5.06	1996	2012	2016	4	0.01	1.17
Bourdieu, 1977	4.97	1977	2001	2012	11	0.01	1.05
Anderson, 1991	4.93	1991	2015	2022	7	0.01	1.05
Latour, 2005	4.9	2005	2010	2013	3	0.00	1.02
Austin, 1962	4.65	1962	2012	2015	3	0.00	1.02

**FIGURE 2:**
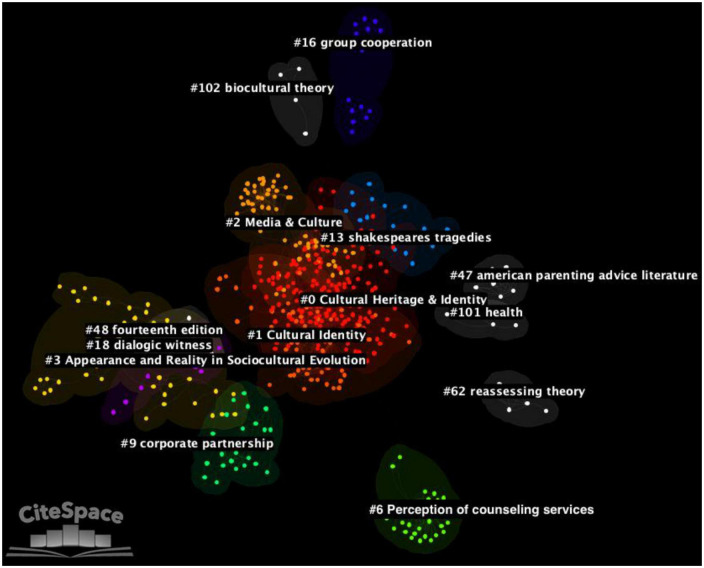
Network of publications generated through the document co-citation analysis (DCA). The 14 major clusters of the network are highlighted by using different colors.

In the network, 14 major clusters of references were identified (see Figure 2 and Table 2 for the complete list). With regards to cluster sizes, the largest cluster, cluster #0, encompassed 184 references (silhouette score = 0.798; average year of publication = 1986), followed by cluster #1 (size = 69 references; silhouette score = 0.938; average year of publication = 1992), and cluster #2 (size = 66 references, silhouette score = 0.943; average year of publication = 1988). The highest values of silhouette scores and therefore of inner consistency were obtained by cluster #62 (silhouette score = 1.000; size = 6; average year of publication = 1991) and cluster #101 (silhouette score = 1.000; size = 4; average year of publication = 1966). On average, almost all major clusters were published in the 20th century and the most recent ones were cluster #16 (average year of publication = 2002; size = 15; silhouette score = 0.995) followed by cluster #13 (average year of publication = 1993; size = 21; silhouette score = 0.981).

**TABLE 2:** Metrics of the 14 major clusters identified with the document co-citation analysis (DCA).

Cluster ID	Size	Silhouette	Mean year
0	184	0.798	1986
1	69	0.938	1992
2	66	0.943	1988
3	46	0.994	1984
6	32	0.988	1959
9	26	0.959	1988
13	21	0.981	1993
16	15	0.995	2002
18	14	0.996	1992
47	7	0.999	1990
48	7	0.996	1987
62	6	1.000	1991
101	4	1.000	1966
102	4	0.997	1992

## Discussion

In the current investigation, a scientometric approach was adopted to examine the impactful publications and trends in literature on appearance and reality. DCA was computed and thematic clusters were identified. Below, we present the contributions of major thematic clusters identified by the Narrative Summary CiteSpace’s function (as in Liu et al., 2020; Lim et al., 2021). We discuss major clusters in chronological order based on the average year in which their documents were published. During analysis, we labeled the clusters to reflect the specific contribution given to the literature on appearance and reality as philosophical, societal, or cultural problems.

### Cluster #6: perception of counseling services

Cluster #6 was labeled as “Perception of counseling services.” In the cluster, the major citing documents were authored by Tan (1967) and Schein (1996), with a coverage–the number of documents in the cluster that were cited by the paper–of 26 and 5 documents, respectively. The body of literature in this cluster closely explored the cultural influences on the preferences and perception of counseling. Take Arkoff et al. (1966) as an example. Results showed that compared to American students, Asian students expressed stronger beliefs that mental health could be enhanced through will power and suppression of unpleasant thoughts. Furthermore, Asian students tended to view counseling as a directive, paternalistic, and authoritarian process. This cluster of work reveals cultural variability in the perception of mental conditions and services. The effectiveness of counseling counts much on clients’ expectations (Bordin, 1955). To many Asian students, the hidden concerns behind counseling services may overwhelm their therapeutic benefits. These concerns may go against the cultural grammar in Asian cultures (e.g., Ji et al., 2010). Likewise, underneath the curse of mental issues or disabilities can be perceived as an opportunity for self-improvement, but only in some cultures but not in others (Chen and Lee, 2021).

### Cluster #3: appearance and reality in sociocultural evolution

Cluster #3 was labeled as “Appearance and Reality in Sociocultural Evolution,” with the major citing documents stemming from Gonlin (2007), Lohse (2007), Tëmkin (2016), and Lloyd (2017) with coverage of 5 documents each. Documents included in the cluster largely examined the sociocultural evolution in humankind (e.g., Gray et al., 2007; Kuhn, 2012; Lloyd, 2017). For centuries, people around the world have pondered the question of what is real and what is not. This question became the staple of philosophy in the West and the East (Ji et al., 2023); it also formed the roots of the rituals and ideologies in classic Mayan societies. Perspectives on this question have varied throughout the long and diverse histories of human societies (e.g., Gossen and Leventhal, 1993; McAnany, 2014). Distinct cultural views on appearance and reality may have to do with how people around the world experience the self. For instance, Markus and Kitayama (1991) noted remarkable cultural differences in the self, relational connectedness and collectivism embraced strongly by many Asian cultures and a sense of agency and individualism embraced strongly by Western cultures. Distinct patterns of the self across cultures have implications for cognition, emotions, and motivations in numerous social contexts (Nisbett et al., 2001; Chiu et al., 2011; Lee et al., 2022).

### Cluster #0: cultural heritage and identity

Cluster #0 was labeled as “Cultural Heritage and Identity.” The major citing documents in the cluster were Markham (2013), Waterton and Watson (2014), and Wilson (2019), with a coverage of 15, 12, and 11 documents, respectively.

Appearance of the past can be a basis of the way people perceive reality of the present. For instance, heritage and collections of historical materials (e.g., museums, heritage sites, and memorials) remind us about the past and, in the process, construct our collective identity and subjective reality in the present, both in a disruptive or an affirming way depending on contexts (Macdonald, 2013). The same applies to journalism (Markham, 2013) and literature, and their depicted stories of (in some cases) fictional characters. For instance, the way in which girl characters have appeared in the literary texts throughout the years has contributed to the collective awareness of what “girls” are or are not as the modern society knows it (Higginbotham, 2013). From the previous cluster, it emerged that the cultural differences in the conception of self-shape the way in which people perceive the world. Research in this current cluster seems to support a bidirectional relationship between the construction of self and the perception of the world.

Discussions on appearance and reality often revolve around a central theme: people make inferences about what they do not see based on what they see. In other words, appearance is an inevitable ingredient in the construction of subjective reality, so vital that it may determine the kind of common sense that people in a community take for granted (Purkhardt, 2015). Examples for this view are vast, including the common intuition that someone is good on the inside if he or she is beautiful on the outside (e.g., Dion et al., 1972; Eagly et al., 1991), or that someone who dresses in pink must be full of feminine qualities, or that someone who appears incompetent must be incompetent in reality (Lee et al., 2020). These questions fall under the broad conceptual umbrella of social constructionism (e.g., Burr, 2015), or the perspective that some knowledge that we embody as objective truth is, in fact, a result of shared ways of thinking collectively developed and reinforced by the group in which we are embedded (e.g., there are certain things that a government is expected to have in appearance to show that it is competent).

Viewing appearance and reality through the lens of social constructionism generates further questions, some of which are non-trivial. For example, it calls for a deeper look at the construction of the self, including how people define, understand, represent, advertise, and socialize themselves in the world (Hallam, 2009). This view resonates with decades of psychological work on the development of self-concepts (e.g., Baumeister, 2022). Sometimes, appearance is a benchmark that people use to justify whether something is real or not. If something looks real, people assume it is. Consequently, the kind of assumptions people have about appearance and reality may shape their reactions to policies, such as whether a bridge should be repainted from time to time for the sake of appearance, even though painting it does not make it any safer. Similar discussions may arise from other societal domains, such as how appearance may be used by some to justify the ownership (e.g., putting up an American flag on the moon) and civil order (Shipton, 2009), or how people react to virtual reality that is built upon intangible information (Geraci, 2010).

### Cluster #2: media and culture

Cluster #2 was labeled as “Media and Culture.” The major citing documents of the cluster were Anderson (2002), Fisher (2010), and Geraci (2010) with a coverage of 34, 10, 7 documents, respectively. A way in which culture shapes people’s perception of the world is through media technologies. The cluster emphasized the role of media and digital technologies in determining what we believe to be reality or appearance. For instance, different news sources often provide multiple versions of the same social issue and they, in turn, influence the public opinion on the problem of interest (e.g., oil spills, environmental risk) (Anderson, 1991).

### Cluster #1: cultural identity

Cluster #1 was labeled as “Cultural Identity.” The major citing documents in the cluster were Kapchan (2014), Dervin (2016), and Howes (2022) with a coverage of 16, 15, and 12 documents, respectively. Appearance guides the perception of reality, of the present, but also of the past and future. Through what can be seen at the moment, people come to understand what was and will be. Similarly to what emerged from the previous clusters of research, appearance and reality are important considerations for cultural identity (Citino et al., 2004; Dervin, 2016), including how people connect to their heritage, how they experience the self under the influence of other cultures, and how they perceive themselves as a member of the global community when the boundaries that once divided cultural groups begin to fade.

Implications for cultural heritage are diverse in scope. For example, would a city that lacks historical buildings be perceived as lacking respect for its cultural roots? Answers might vary, depending on how appearance is perceived as a reflection of reality. Similar questions apply to other forms of cultural heritage, from music (Brandellero et al., 2014), costume (Kuper, 1973), language (Baker, 2015; Kumar, 2016), monumental architecture (Tsukamoto and Inomata, 2014). Rituals (e.g., is it a threat to cultural identity if someone holds a wedding that deviates from cultural traditions?) and art, too, deserve equal discussions as both are foundational elements of culture (Kapchan, 2014). Moving forward, research can address the impacts of globalization (King, 2016) on cultural identity (Citino et al., 2004), intercultural competence (Dervin, 2016), nationalism (Hunt, 2016), all of which may decorate the literature with new theoretical flavors through the lens of appearance and reality.

## Limitations

While the results of the current scientometric review are informative, limitations are in place, with some being intrinsic to the scientometric method (see also Carollo et al., 2021a,b,c). First, the results depend on the downloaded sample of documents and the platform from which the data pool was retrieved (i.e., Scopus). Thus, the insights obtained from the present review might be extended, such as by adopting different keywords in data search or downloading data from different platforms (e.g., Web of Science). Other limitations are intrinsic to the approaches to systematic reviews that strongly rely on patterns of co-citation between documents. While the scientometric approach allows to investigate a large data pool of document in a data-driven way, patterns of co-citation are only examined under a quantitative point of view, and not qualitative. In other words, the reasons behind citation patterns are neglected in favor of a quantitative approach. Also, relying on patterns of citation between documents strongly biases the analysis in favor of old publications rather than more recent ones. In fact, old publications may emerge as more impactful only because they have had more time to obtain citation counts compared to recent publications, and not necessarily because they are more impactful intellectually. Nonetheless, even when the aforementioned limitations are considered, the scientometric approach has been doing the scientific literature a great service by establishing systematic reviews in the fields of neurobiology, social neuroscience, and clinical psychology, to name a few (Carollo et al., 2021a,b,c; Lim and Aryadoust, 2021; Lim et al., 2022).

## Conclusion

Through a scientometric approach, the current work examined the literature on the assumptions about appearance and reality. A sample of 4,771 documents including 282,591 cited references was analyzed with CiteSpace software. Both impactful documents and thematic domains of literature were identified and discussed. Specifically, the way people associate appearance with reality seems to matter in all walks of life, cutting into domains of social constructionism, heritage and memory, cultural identity, and other social domains (Lee et al., 2020; Ji et al., 2023). These findings suggest that appearance and reality, as theoretical constructs, are not exclusive to only one or two life domains. Rather, these constructs are diverse in scope and forms, penetrating numerous historical, contemporary, and cultural contexts. Moving forward, researchers can consider integrating the present work with theoretical frameworks in their own fields, such as frameworks on the self (Markus and Kitayama, 1991) and thinking styles (Nisbett et al., 2001) in cultural psychology (see also Lee et al., 2020; Ji et al., 2023).

## Data availability statement

The original contributions presented in this study are included in the article/supplementary material, further inquiries can be directed to the corresponding author.

## Author contributions

AC, AL, and GE: conceptualization. AC, AS, and GG: data curation. AC and AS: formal analysis. AC: investigation. GE: supervision. AC, AS, SF, and AL: writing—original draft. All authors writing—review and editing, read, and agreed to the published version of the manuscript.
